# Draft genome sequence of propane- and butane-oxidizing *Rhodococcus ruber* IEGM 333 able to accumulate cesium

**DOI:** 10.1128/mra.00101-24

**Published:** 2024-03-28

**Authors:** Irena Ivshina, Maria Kuyukina, Anastasiia Krivoruchko

**Affiliations:** 1 Perm Federal Research Centre, Perm, Russia; Rochester Institute of Technology, Rochester, New York, USA

**Keywords:** propanotrophy, *Rhodococcus ruber*, cesium accumulation, gas prospecting

## Abstract

A genome of *Rhodococcus ruber* IEGM 333 was sequenced and annotated. This bacterium had pronounced propane- and *n-*butane-oxidizing and cesium-accumulating activities. The obtained sequence could be used to reveal the genetic mechanisms of these activities and efficiently exploit the biotechnological potential of propanotrophic *Rhodococcus*.

## ANNOUNCEMENT

**Fig 1:**
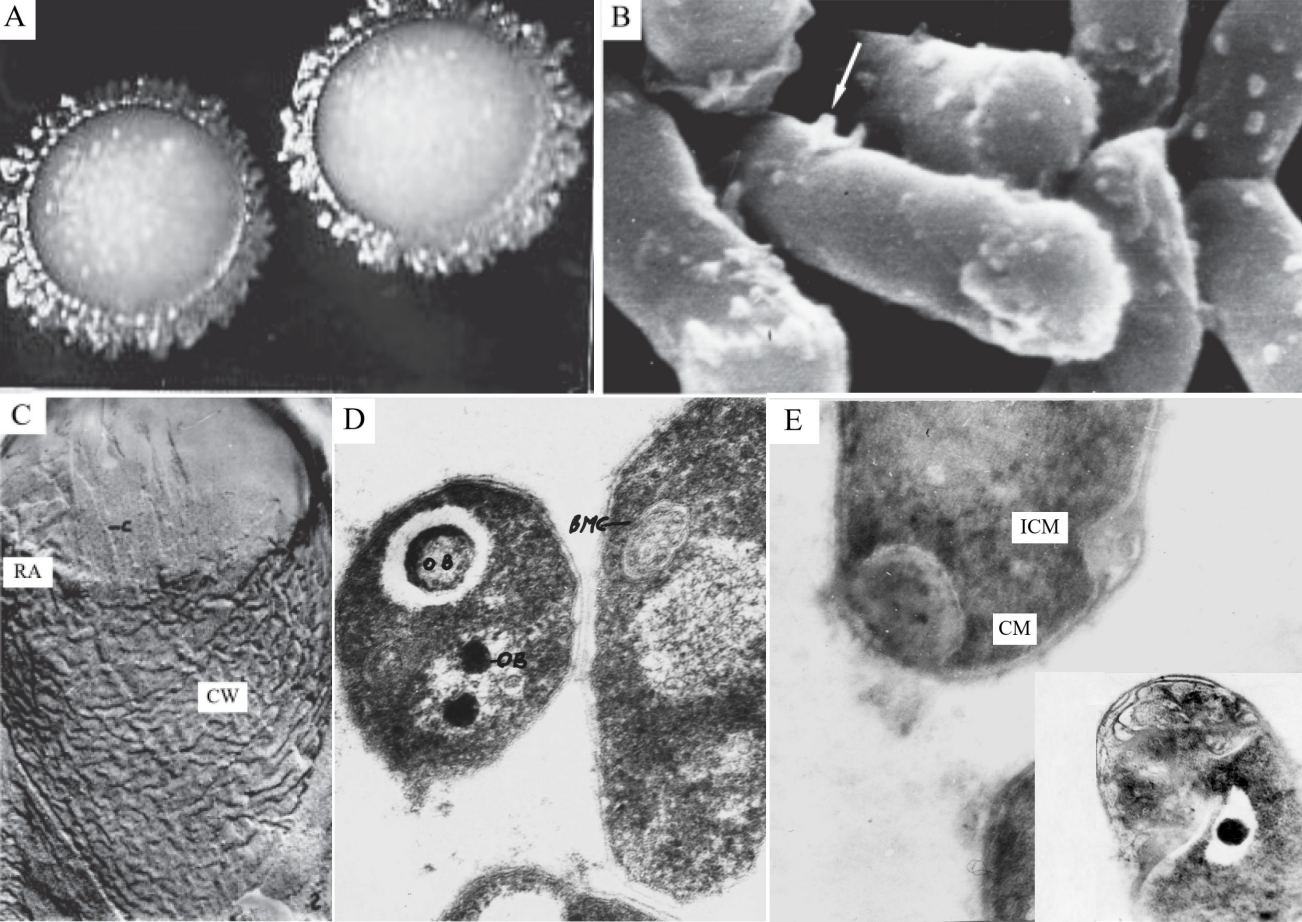
Morpho-functional changes in *Rhodococcus ruber* IEGM 333 cells grown in the propane atmosphere. (A) Colonies on mineral agar after incubation in a gas mixture propane:air 1:4, magnification ×36. The colonies have tree-like dry filaments along their edges for anchorage on the substrate, which also occurs due to the ingrowth of cells into the substrate. (B) Cells under a scanning electron microscope, magnification ×44,000 (the arrow shows cell surface appendages of the diameter ≥40 nm, which are invisible under a light microscope). These multiple strobilaceous appendages are responsible for contact of cells with each other, communications between contacting cells, and an efficient generation of “a cooperative cellular system” being a response to the propane as a limiting growth substrate. (C) The fracture surface of a cell was obtained using the cryo-fractography, magnification ×67,000 (CW—the cell wall; and RA—a ringed protrusion at a cell end). D—Ultrathin sections of cells, magnification ×80,000 (ОВ—intracellular inclusions, which are volutin granules (smaller electronic-dense inclusions) and vacuole-like formations, filled with a structureless substance, surrounded by an osmiophilic ridge, and acting as specialized compartments for propane oxidation (a big electron-dense inclusion); ВМС—a well-developed intracytoplasmic membrane system in the form of densely packed vesicles, occupied a big part of the space at the periphery of the cytoplasm, increasing the total surface of the cytoplasmic membrane, and functionally bound with the metabolism of propane oxidizing cells; remarks are translated from Russian). (E) Ultrathin sections of cells with visible invaginations of the cytoplasmic membrane, magnification ×45,000 (CM—the cytoplasmic membrane; and ICM—an invagination of the cytoplasmic membrane, or a proliferation of the cell wall material into the cytoplasm, separated from the protoplast with the cytoplasmic membrane). The cell wall proliferation is enhanced at the growth of cells in the presence of *n-*butane (**C_4_**). The extra growth of the cell wall, which increases its total surface, results in the generation of a specialized depository for full saturation of the cell with propane. The cell wall and its surface are basic places for the accumulation of gaseous *n-*alkanes. All detected cellular changes disappear, and cells return to their previous phenotype after repeated passages and incubation in nutrient broth. Modified with permissions from references (9, 10).

The ability to use higher gaseous homologs of methane, namely propane and *n-*butane, as growth substrates is a rare metabolic feature of prokaryotes (1). This type of metabolism is confirmed, for example, for a few *Rhodococcus* species (*R. aetherivorans*, *R. opacus*, *R. rhodochrous*, and *R. ruber*) (2, 3), *Thauera butanivorans* (4), and *Arthrobacter* sp. PG-3-2 (5). Propane and butane degraders can be successfully used in the prospecting for natural gas deposits; and the unique enzymes necessary for C3 and C4 alkane assimilation provide propanotrophs with additional molecular tools for attacks on emergent and recalcitrant organic pollutants (3, 6). The propane- and *n-*butane-oxidizing *Rhodococcus ruber* strain IEGM 333 (Fig. 1) was isolated from underground water in an oilfield outline zone, Perm region, Russia, and deposited in the Regional Specialized Collection of Alkanotrophic Microorganisms (acronym IEGM, number 768 WDCM, http://www.iegmcol.ru/strains/rhodoc/ruber/r_ruber333.html). This strain accumulated more than 60% of cesium ions from the medium at their concentration of 0.2 mM (7) and also transformed 24% β-sitosterol to androstane derivatives at its concentration of 500 mg/L (8). These activities can be used in the treatment of wastewater contaminated with radionuclides and the synthesis of intermediates for hormonal drugs (7, 8).

For DNA extraction, the *R. ruber* IEGM 333 cells were recovered from a lyophilized culture after storage for 6 years and grown in LB broth at 28°C and 160 rpm for 28 h. DNA was isolated using the MagMAX Microbiome Ultra Nucleic Acid Isolation Kit (Thermo, USA) and a KingFisher Flex automatic station (Thermo Fisher Scientific) following the manufacturer’s protocol. A paired-end library [2 × 100 nucleotides (nt)] was produced using Illumina DNA Prep and sequenced on an Illumina NovaSeq 6000 instrument. The quality of the library was estimated using a fluorescence analysis and a 2100 Bioanalyzer (Agilent Technologies). A total number of 81,187,404 reads was obtained. Demultiplexing of the sequenced reads was performed with Illumina bcl2fastq (2.20). Adapters were trimmed with Skewer (version 0.2.2) (11). The quality of the FASTQ files was analyzed with FastQC (version 0.11.5-cegat) (12). The data were assembled with SPAdes v. 3.14.1 (13) at the contig level. The assembly consisted of 147 contigs with a total sequence length of 5,601,287 bp, an N50 value of 103,960 bp, a GC content of 70.5%, and coverage of 650×. Average nucleotide identity (ANI) and digital DNA/DNA hybridization (dDDH) values were calculated using ANI Calculator (https://www.ezbiocloud.net/tools/ani) (14), Genome-to-Genome Distance Calculator 3.0 (https://ggdc.dsmz.de/ggdc.php#) (15), and Type (Strain) Genome Server (https://tygs.dsmz.de/) (15, 16). The highest OrthoANIu and dDDH values of 99.16% and 95.4%, respectively, were obtained for *R. ruber* (NCBI: txid 1830). The annotation of coding sequences (CDS) was performed using PGAP 6.5 (17). Default parameters were used for all software unless otherwise specified.

A total of 5,193 CDS, 5,115 CDS with protein, and 52 RNAs were found in the *R. ruber* IEGM 333 genome. A total of 33 monooxygenase genes including 1 methane (propane) and 2 alkane-1-monooxygenase genes and 53 metal/cation transporter genes including 12 CDS coded for potassium transporters (potentially responsible for transport of cesium) were found among annotated CDS.

## Data Availability

This Whole-Genome Shotgun project has been deposited in DDBJ/ENA/GenBank under the accession numbers JASHLC010000001-JASHLC010000147. The version described in this paper is the first version available at https://www.ncbi.nlm.nih.gov/nuccore/JASHLC000000000.1/. Raw sequence reads were deposited in the Sequence Read Archive with accession number SRR27720759 under BioSample accession number SAMN35084007 and BioProject accession number PRJNA783162.
